# Evaluation of Ventilation-Induced Lung Inflammation Through Multi-Scale Simulations

**DOI:** 10.1109/JTEHM.2018.2795031

**Published:** 2017-12-27

**Authors:** Israr Bin M. Ibrahim, Ramana M. Pidaparti, Kevin R. Ward

**Affiliations:** College of EngineeringUniversity of GeorgiaAthensGA30602USA; Michigan Center for Integrative Research in Critical CareUniversity of MichiganAnn ArborMI48109USA

**Keywords:** Human lung, airway inflammation, multi-scale model, finite element analysis, cellular automata

## Abstract

Ventilation-induced lung injury is a common problem faced by patients with respiratory problems who require mechanical ventilation (MV). This injury may lead to a greater chance of developing or exacerbating the acute respiratory distress syndrome which further complicates the therapeutic use of MV. The chain of events begins with the MV initiating an immune response that leads to inflammation induced tissue material alteration (stiffening) and eventually the loss of lung resistance. It is clear from this sequence of events that the phenomenon of ventilation induced injury is multi-scale by nature and, hence, requires holistic analysis involving simulations and informatics. An effective approach to this problem is to break it down into several major physical models. Each physical model is developed separately and can be seen as a component in a larger system that comprises the scale of the problem being investigated. In this paper, a multi-scale system consisting of breathing mechanics, tissue deformation, and cellular mechanics models is developed to assess the immune response. To demonstrate the potential of the model, a fluid–solid model is employed for breathing mechanics, a plane-strain elasticity model is applied to assess tissue deformation, and a cellular automata (CA) model is developed to account for immune response. A case study of three lower airways is presented. The CA model shows that this increased the immune response by five times, which correlates with alteration in the tissue microstructure. This alteration in turn is reflected in the material constant value obtained in the tissue mechanics model. However, the changes in strain rates in the airways after inflammation (and hence, lung compliance) were not as significant as the rates of change in immune response. Finally, results from the fluid–solid model demonstrate its potential for airflow characterization caused by tissue deformation that could lead to disease identification.

## Graphical abstract

Multiscale simulations illustrate the inflammatory response under mechanical ventilation induced stretch in lungs.

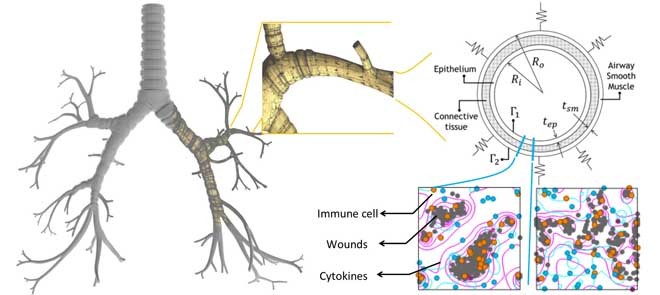

## I. Introduction

Despite on-going efforts to reduce the risk of injury associated with Mechanical Ventilation (MV) application, it is still a major issue that contributes to mortality in patients with respiratory problems [1]. Positive-pressure MV devices are commonly used by specialists and they increase pressure inside the alveoli well above normal lung function levels. This excess pressure leads to various injuries from different mechanisms; some injuries are caused by failure to maintain elasticity criteria, while others result from biochemical signals leading to inflammation. It is also alarming to note that these injuries do not stop at the pulmonary airways, but are able to spread to other organs through biochemical signaling. Ventilation induced injury is essentially the result of a chain of events that unfold as a side effect of excessive pressure. It is therefore imperative that we understand the underlying mechanisms of this multi-scale event that leads to injury. Various in vivo, in vitro and in-silico models have been developed to explain partial and localized aspects of the injury and inflammation. According to previous studies, low peak airway pressure (14 cm H2O) does not lead to histological changes of damage in the lung, while higher ventilation pressure (30 cm H2O) results in mild perivascular edema. In addition, ventilation at 45 cm H2O (without PEEP) would lead to severe hypoxia and mortality in less than one hour [2], [3]. Similar findings have been observed with ventilation in small animals. A high peak inspiratory pressure for just 2 minutes is sufficient to induce pulmonary edema in small animals while larger animals require much longer periods of ventilation for changes to be evident [4], [5]. Prevention of ventilator-induced lung injury initiation would be possible by keeping transpulmonary pressure within the physiological range. The prone position may attenuate ventilator-induced lung injury by increasing the homogeneity of transpulmonary pressure distribution [6].

Building upon findings from both in vivo and in vitro studies, the in-silico model has been used to gain additional insights into the lung injury. The lung as an organ works essentially like that of a medieval forge blower; modulating pressure by regulating the volume of the air container (the chest). Hence, various fluid-based models have been developed, including lumped parameter models and fluid-solid models. A major finding from computational models indicates that different airflow characteristics are obtained by altering airway properties and pressure drop characteristics in diseased lungs with obstructed airflow. Several computational studies have been completed to date including the following: measurement of tissue parameters with a system of airway generation identification (Lipsett, J., 2002), airflow patterns and airway wall stresses in the first generations of lower airways in a real lung geometry with moving wall [7] and rigid walls [8], influence of laminar and turbulent airflow throughout the entire respiratory tract on air flow [9] and link between the distribution of airflow to local parenchymal inflation and information about local tissue strains and stress elevations [10].

On the other hand, it is known that the tissue possesses hyper-elasticity, and is sensitive to mechanical stimuli. Various non-linear deformation, remodeling and growth models have been developed. Meanwhile, to gain a deeper understanding of microscopic dynamics, the cell population in the tissue needs to be modeled. This model would constitute a series of equations to describe cell behavior and cytokines kinetics, which could be cumbersome if one had to account for spatial aspects. Hence, many researchers have turned to discrete models to study this type of system. Brown *et al.* [11] created an agent-based model to examine the role of macrophages and fibroblasts in the inflammatory and fibrotic response to particulate exposure. They suggested that the mechanisms in this in silico model might be expanded to serve as a platform for the investigation of the processes of inflammation and fibrosis that result from particulate exposure in the lung. Reynolds *et al.* [12] developed a CA model for pulmonary inflammation. Their results showed the influence of high inflammation on healing, persistent infection, and resolved infection. Dutta-Moscato *et al.* [13], created an agent-based model (ABM) of liver tissue in order to computationally examine the consequence of liver inflammation. They concluded that a computational model of liver inflammation on a structural skeleton of physical forces could recapitulate key histopathological and macroscopic properties of carbon tetrachloride-injured liver. An and Kulkarni (2015) [14] used an inflammation and cancer agent-based model to bridge the gap between basic mechanistic knowledge and clinical/epidemiologic data. Their results showed that increasing inflammatory environments leads to damaged genomes in microtumors. Implementation of qualitative behavior from observation into computational modeling is one of the discrete model’s key features. Discrete methods such as Cellular Automata (CA) generally address evolution of the state of components (such as substance A converts into substance B) instead of evolution of a variable with respect to real time. Hence, the CA is a powerful tool to deal with complex system spanning wide temporal scales.

Various pulmonary inflammation processes are phenomena that span across diverse spatial and temporal scales. An in-silico model that accounts for such diverse aspects is therefore critically needed. A computational analysis should not stop at a particular scale level to obtain a holistic picture of the entire system. To achieve this, one can couple multiple scales into one or a series of equations, or sequence several levels of a model, as previously demonstrated [10]. Another approach is to isolate the scale of physics by assuming that each physical model developed works separately as part of a larger system. This paradigm derives from the system modeling perspective, where each model acts as if it is a component or “port”. In the subsequent discussion, this paradigm is presented to analyze a simple case of ventilation-associated inflammation.

## II. Materials and Method

### A. Multi-Scale Approach

**FIGURE 1.**
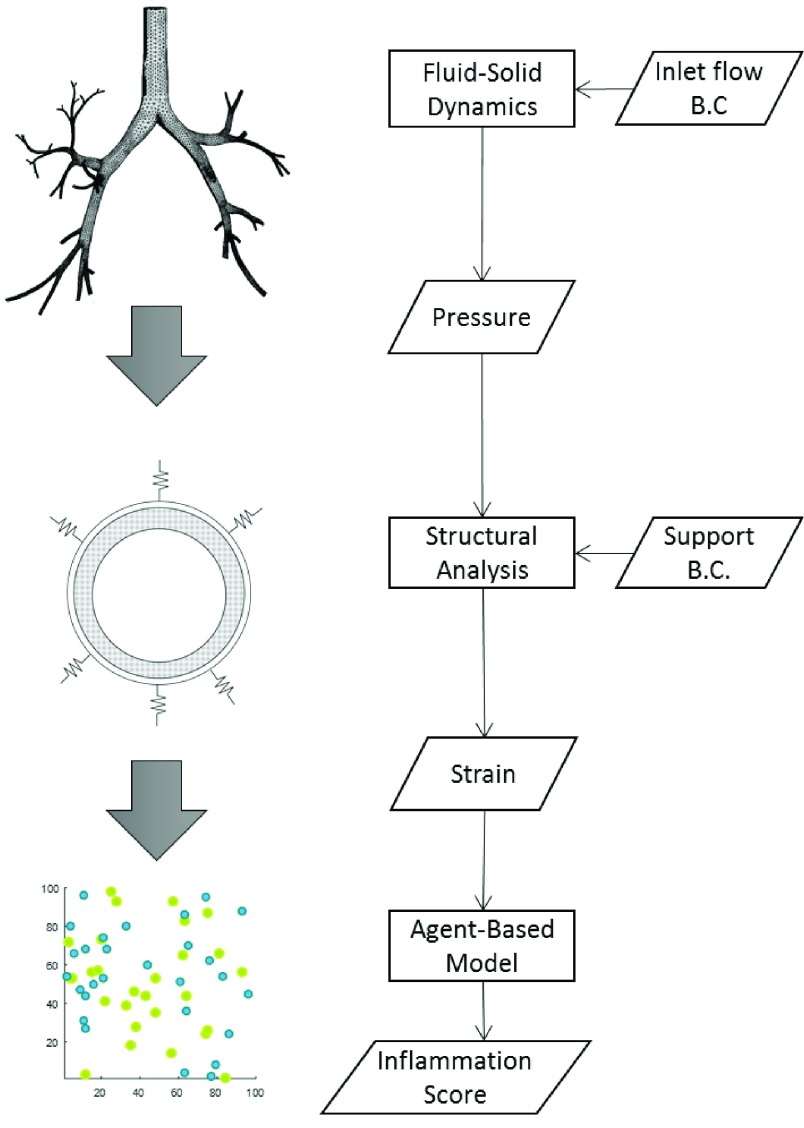
Flow chart of multi-scale simulation.

The inflammation occurring during mechanical ventilation is an event that needs to be investigated holistically through multiple spatial and *temporal* scales. A way to approach problems of this nature is to break down the phenomenology to its components. Figure 1 shows the proposed components of ventilation-induced inflammation. Each component (circle) consists of its own analysis. In the next section, the analysis for each component is presented and explained.

The model developed in this study is aimed toward understanding the cascade of events involved in inflammation that starts with the pulmonary tissue and ends up influencing lung resistance. There are many models developed for each component as shown in Figure 1. Since the multi-scale approach is presented as a system passing and receiving information, the computational model for each component can be added and modified. In this study, we present the computational models developed under the multi-scale approach, along with the rationale for choosing the models.

### B. Cellular Scale

The pulmonary tissue is constantly under stretch (and over-stretch in the case of Mechanical Ventilation). Stretching tissue induces mechano-biological responses. The tissue itself can be seen as a microenvironment for a congregation of cells. Immune cells are regulated in connective tissue to respond to damage and foreign matter that threaten the tissue. The immune cells will be activated by various stimuli to initiate its function as an immune system. Stretch experienced by the tissue is a mechanical stimuli that activates immune cells. The stretch itself can be seen as an elastic field on tissue space. Epithelial cells make the outer layer of tissue, which is the interface between tissue and surrounding environment. In this study, the number of death epithelial cells will be used to estimate inflammation severity.

**FIGURE 2.**
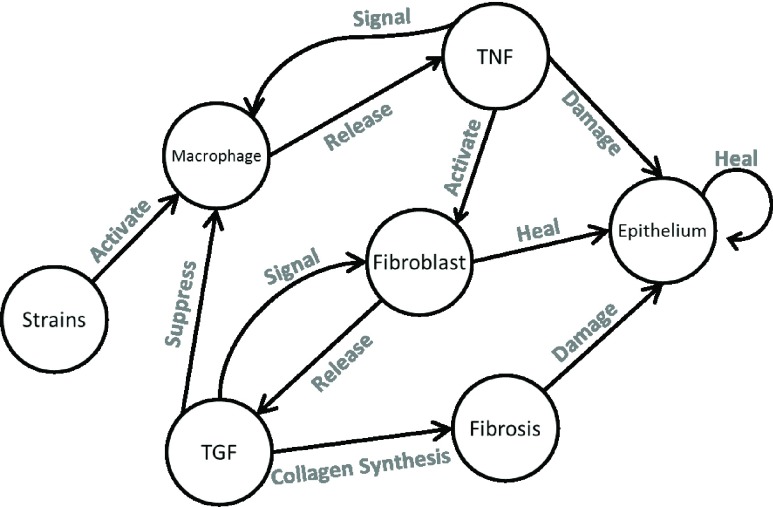
Based on strain from tissue scale simulation, an agent-based model is used to simulate the inflammatory response.

Since there are many interactions involved in this kind of system, an agent-based model is an appropriate modeling tool. The tissue constituents may exhibit a diverse array of interactions as a result of mechano-biological responses. In this study, we focus on innate immune response triggered by mechanical stretch. A network of interactions is proposed as shown in Figure 2.

Stochastic models based on interaction of agents have been used to model liver [13] and inflammation by particulate matters [15]. Our previous study presented an agent-based model for stretch-induced inflammation, aimed for airway tissue [16]. The model consists of agents divided into three categories: fixed cells (epithelium), wandering cells (macrophages and fibroblasts) and cytokines (TNF and TGF). The agents occupy a discrete two-dimensional space, with fixed neighborhood. We summarized the rules as described in [16], as below:

1) Epithelium have three state, alive, fibrosis and dead. “Heal” action (as in Figure 2) changes an epithelial cell state to “alive”, and “damage” action changes its state to “dead”. The probability of state transition to “damage” is determined by concentration of TNF, and the probability of state transition to “alive” by “heal” is determined by concentration of TGF.
2) Wandering cells exhibit random walk. However, the probability to move to adjacent location is determined by cytokines. Hence, the randomness of the walk is not uniform. This rule represents chemotaxis.
3) Cytokines are released by wandering cells. TNF is released by macrophages and the probability of TNF release by macrophage is higher when TGF concentration is low.
4) Similarly, TGF is released by fibroblast and the probability of TGF release by fibroblast is higher when TNF is higher.
5) Cytokines diffuse according to diffusion equation,
  \begin{equation*} dC/dt=D\nabla ^{2}C-K.C \end{equation*}
  where $\nabla ^{2}$ is Laplacian, $C$ is concentration, $D$ is diffusity, *and* ${K}$ is a constant determining the rate of disintegration.
6) Each location occupied by the cells above has a value of strain. This value may fluctuate or static.
7) Each state transitions as in Fig 2 is probabilistic.

Each agent executes rules assigned on them in the two dimensional space that represents airway tissue section. Figure 2.1 illustrates the model in which the wandering cells are visible. The dimension of the two-dimensional space and related parameters of the model used in this study is shown in Table 1.

**TABLE 1:** Cellular Scale Model Parameters

Parameters	Value
Dimension	100 $\times$ 100
Ratio of TNF to TGF diffusity	0.7
Ratio of TNF to TGF disintegration constant	0.1
Ratio of wandering cell’s velocity to dimension	0.03
Ratio of wandering cell’s population (each type) to dimension	0.0025

The stochastic model has been shown that certain strain level leads to a typical inflammation course time, where immune response quickly rises early, then slowly decreases, resembling a skewed normal distribution. The model dynamic is based on probability but results of each trials have been shown to have low variance [16].

### C. Tissue Scale

The main focus in soft tissue analysis is the deformation of thin soft material, specifically in circular form. It has been shown that the non-linear deformation of airway tissue leads to non-linear buckling as a means for the tissue to accommodate energy from loading [17]. Of main interest in this study is the reduction in lung airway compliance, or the ability to inflate or dissipate energy inserted by air pressure. The airway tissue under inflammation will alter its mechanical properties. This alteration is caused by rearrangement of tissue microstructure during the dynamic event of inflammation, as will be discussed in a later section. The most important variables in tissue mechanics model are tissue thickness and elastic properties [18].

The stress experienced by an elastic body can be modeled by,

\begin{equation*} \nabla \sigma =\rho _{s} \partial ^{2}u/dt^{2} \end{equation*}

where $\nabla $ is gradient, $\sigma $ is Cauchy stress, $\rho _{\mathrm {s}}$ is solid density, $t$ is time and $u$ is displacement. The boundary conditions for this case are as follow. Boundary $\Gamma _{1}$ (epithelium) was subjected to pressure acting on normal direction of the boundary. Boundary $\Gamma _{2}$ was supported with spring, with spring constant of 600 N/m^2^. Elastic support was used to model airways tethering to alveolar sacs surrounding them. Its value was adopted from [8].

The model as described so far is a multi-region model. The three regions are: epithelium, connective tissue and airway smooth muscle (ASM). This scheme of categorization can also be found in [17]. The two inner regions (the epithelium and connective tissue) were modeled as Neo-Hookean material. The strain energy density of Neo-Hookean elasticity is defined as,

\begin{equation*} W=(\mu /2).(\text {I}_{1} -3)+(1/\text {d}).(\text {J}-1)^{2} \end{equation*}

where $I_{1}$ is deviatoric first principal invariant, $J$ is Jacobian, $\mu $ is incompressibility parameter and d is incompressibility parameter. The latter two are material parameters of this material model. The outer region (ASM) was modeled as linear elastic. The materials parameters for the three regions are given in Table 2. Following [19], it is assumed that the inner layer has Neo-Hookean shear viscosity of $\mu =5$ MPa, and the ratio of elastic properties between inner and middle layer is initially 10. The model was solved through Finite Element Method using ANSYS Workbench 2016, similar to our previous study [20]. Based on the cited study, the mesh with 800,000 elements is enough to reach convergence.

**TABLE 2:** Material Constants Used in Tissue Scale Model

Tissue type	Material Constant	Note
Epithelium	$\mu =5$ MPa, $D_{1}=5000$ MPa^−1^	Neo-Hookean
Connective Tissue	$\mu =0.55$ MPa, $D_{1}=5000$ MPa^−1^	Neo-Hookean
ASM	$E=1$ MPa	Linear Elastic

Each of three region has their own morphological characteristics at each generation of airways. Table 3 shows morphological characteristics of bronchioles based on [21].

**TABLE 3:** Airway Morphological Characteristics

Generation	Lumen Diameter (cm)	Airway Smooth Muscle thickness (cm)
G6	0.232	0.0013
G7	0.1947	0.0012

### D. Organ Scale

Breathing primarily involves regulation of airflow in the pulmonary tree. Therefore, the pulmonary tissue is being constantly subjected to pressure swings. A number of models based on fluid dynamics have been used. The lumped parameter method has been used to quantify airflow through the principle of conservation of flow [9], [22] and to eventually classify the type of flow dynamics for different conditions such as obstructive lung disease. The lumped parameter method is efficient and reveals useful insights into airway dynamics, but it bypasses certain physical characteristics that could be useful in analysis. To achieve this, a more elaborate analysis is needed.

Fluid-solid interaction analysis has been established over the years with the introduction of FEM analysis and moving mesh method [23]. In this approach, the airway tree can be treated as an elastic structure that is under constant oscillating strain by airflow during breathing. There are two common cases of breathing: normal breathing where the airflow is allowed by regulating intrathoracic pressure, and mechanical ventilation where air is injected into the lung. We consider a case where air is injected via mechanical ventilation. The airflow at the end tube of the lung (the inlet) is injected in a specific way and can be represented by an exponential wave.

**FIGURE 3.**
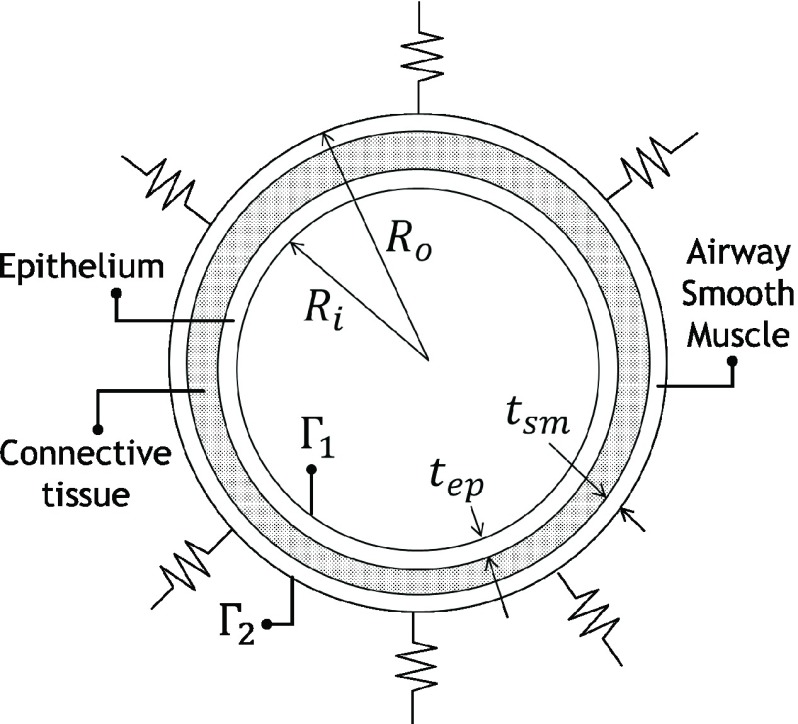
At tissue scale, structural analysis was employed. The radial strain of airway tissue is of interest hence a cross section model of airway tissue was considered. The boundaries of the model are denoted as $\Gamma _{1}$ and $\Gamma _{2}$. The elastic supports on $\Gamma _{2}$ are shown as a series of spring attached along $\Gamma _{2}$.

Figure 3(a) shows an illustration of the problem. The mechanical ventilation inflow can be modeled using equations as shown in Table 3. The Navier-Stokes equation can be used to describe the dynamics of fluid,

\begin{align*}&\text {Continuity: }\partial \rho /dt+\nabla.(\rho.v)=0 \\&\text {Navier-Stokes: }\rho (\partial v/dt+v.\nabla v)=-\nabla p+\nabla.\tau \end{align*}

where $\nabla $. Is divergence, $v$ is fluid velocity, $p$ is fluid pressure, $\rho $ is the fluid density, and $\tau $ is a constitutive relationship that relates fluid’s dynamic viscosity (material constant) and fluid velocity. In this case, the fluid is air and hence air viscosity of $1.79 \times 10^{\mathrm {-5}}$ kg/(m.s) was used.

Equation (2) describes the dynamics of an elastic solid. To model the fluid-solid interaction during breathing, a three-dimensional organ model was constructed and this model was imported into the computational fluid-solid package in ANSYS Workbench 2016.

**FIGURE 4.**
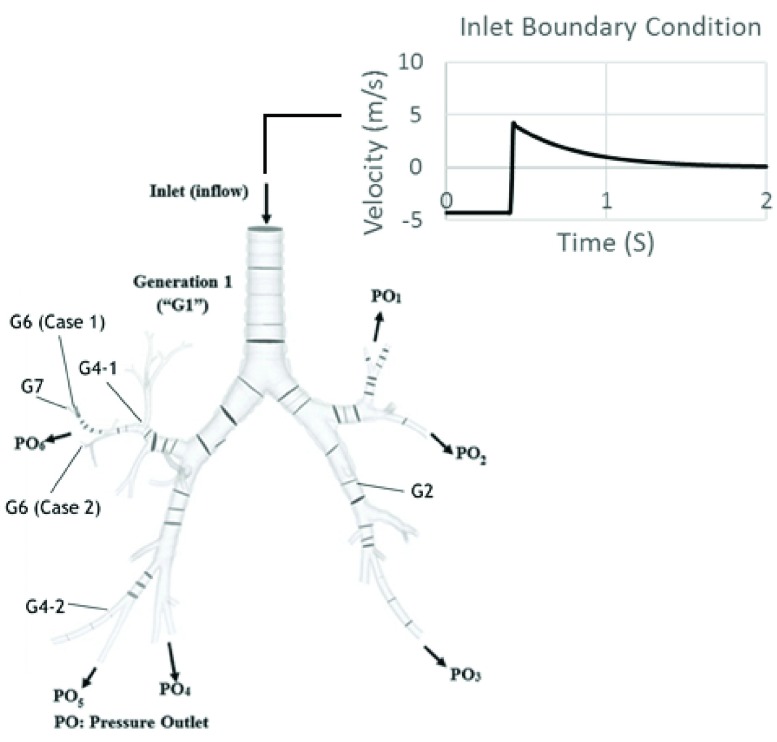
Organ scale model and its inlet boundary condition. The location of pressure outlet boundary conditions are also shown here.

Figure 4 shows the completed model up to generation 7. Following our previous study [24], the mesh was determined to be 1,034,392 elements for the fluid model, and 444,462 elements for the solid model. The meshes were found to be sufficient to reach convergence [24].

The organ model’s boundary condition was an in-flow at the inlet (as shown in Fig 4). It was modeled using two equations as shown in Table 4. The equations represent inspiration and expiration, where $Q$ is flow rate, *GN* is generation number, $T_{in}$ is inspiration time, $T_{EX}$ is expiration time, and $S$ is airway cross-sectional area. Inspiration and expiration time were chosen to be 0.4 s and 2.0 s, respectively. Flow rate is equal to the proportion of tidal volume to the inhalation time. Tidal volume is the lung volume that represents the normal volume of air displaced between inhalation and exhalation when extra effort is not applied. The tidal volume here is set to be 420 ml.

**TABLE 4:** Organ Model’s In-Flow Boundary Conditions

Portion of breathing	Equation
Inspiration ($T \,\, < \,\, T_{IN }$)	$Q/(S\times 2^{GN-1})$
Expiration ($T > T_{EX}$)	$-Q\times e^{(-(T_{IN} +T_{EX})/T_{EX})}/(S\times 2^{GN-1})$

The boundary conditions for structural analysis are as follow. The pressure outlets (Fig 4) were assigned as fixed support. The lumen of the airways was subjected to pressure computed from the FSI. The material model at the organ model was determined to be elastic with Young’s modulus of 99 MPa, a typical linear elastic Young’s modulus for tissue [8].

## III. Results and Discussion

Organ scale model is used to compute the pressure acting on airway tissue by breathing. The tissue model then computes strain experienced by tissue. Lastly, agent-based model was used to simulate inflammatory response by tissue constituents.

**FIGURE 5.**
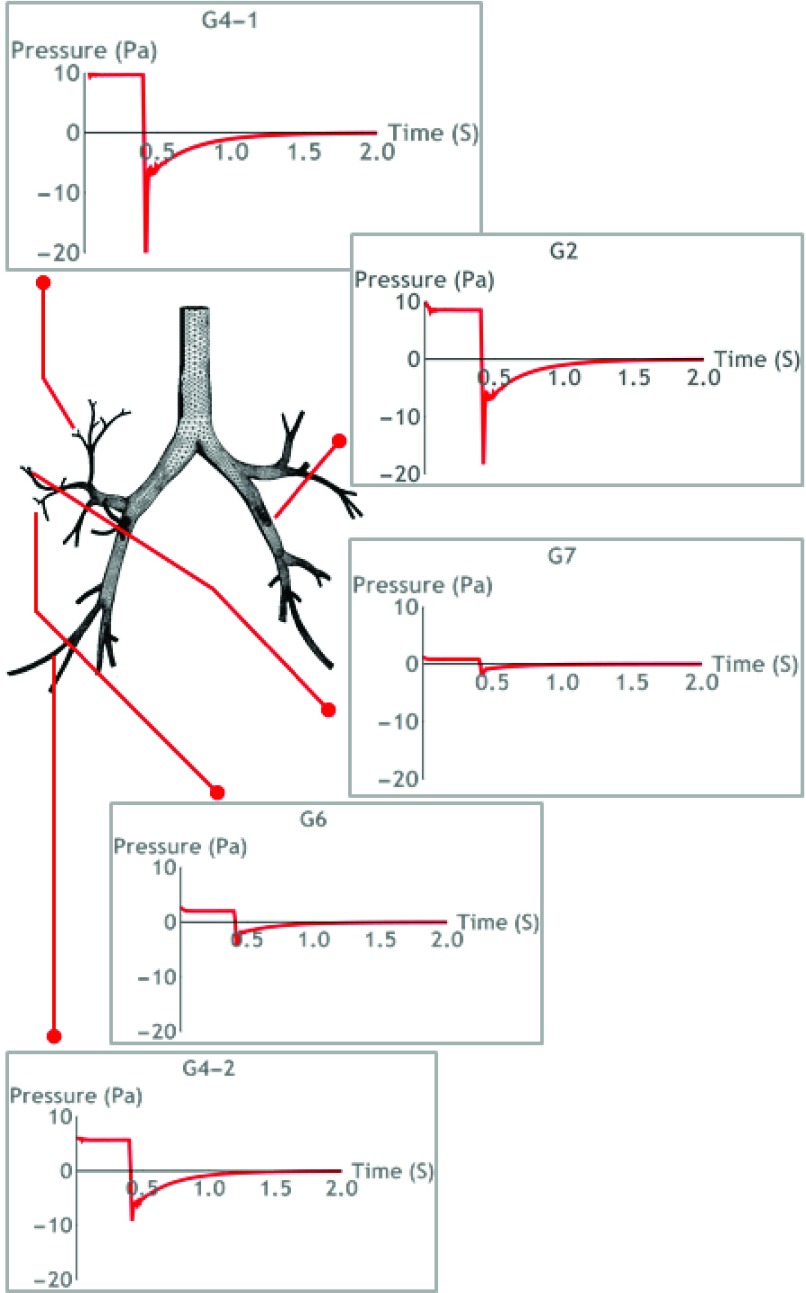
Pressure on various location of airways based on organ-scale simulation.

From the organ scale model, pressures acting on tissue were presented in Figure 5 for several airways. As can be seen, the pressure acting on G2 and G4-1 airways are the highest: pressure drops over generation can clearly be observed here [25]. We present an example case to analyze the effect of pressure down to cellular level.

**FIGURE 6.**
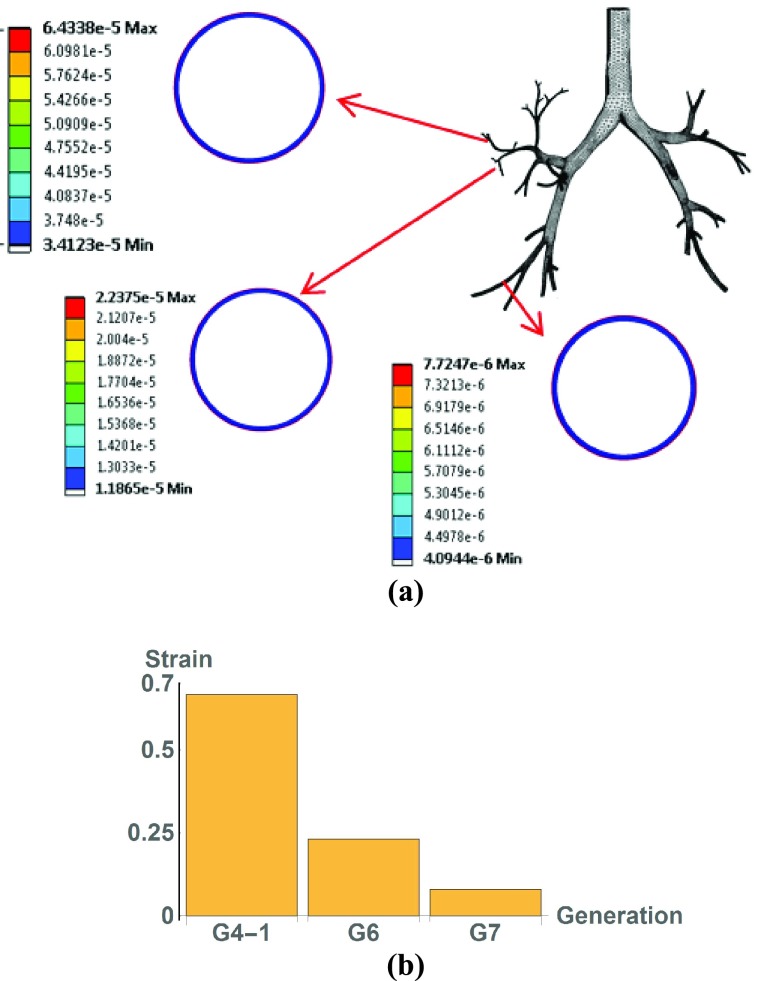
Strain at three selected airways for cell-scale simulation, (a) locations, (b) strains caused by pressure.

Figure 6 shows three selected airways, namely G4-1, G6 and G7. The strains experienced by airway tissue are shown in Figure 6(b). As can be seen, airway G4-1 experienced the highest strain.

**FIGURE 7.**
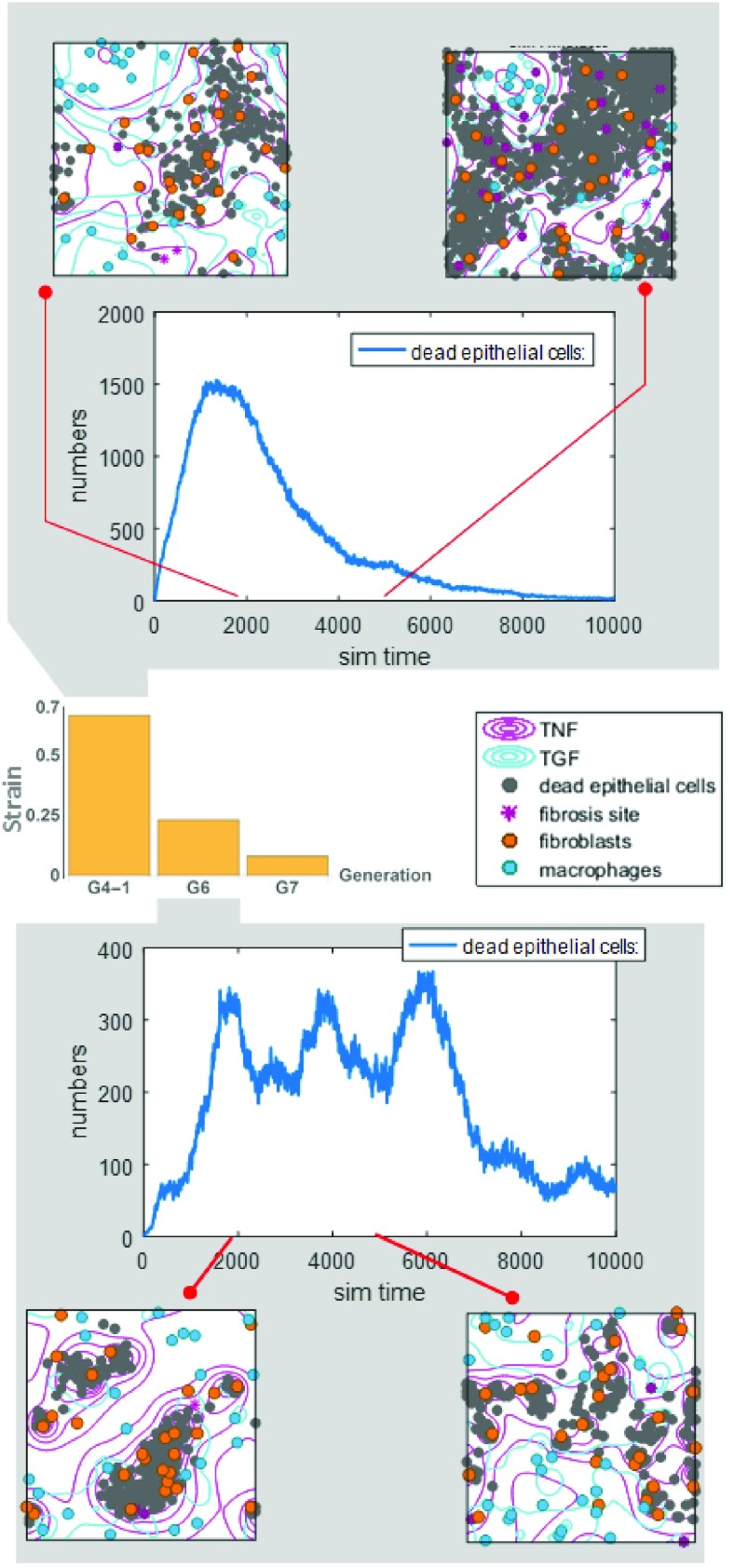
Various inflammatory responses are obtained from cellular scale simulation. The count of dead epithelial cells has been taken as the level of inflammatory response. The figure also shows captures of tissue constituents (fibroblasts and macrophages) locations at several time step.

Figure 7 shows inflammatory response as simulated by agent-based model at cellular scale. As can be seen, there is no inflammatory response in G7, with since it experienced low strain. The tissue constituents exhibit random walks in this case. G4-1 exhibited the highest inflammatory response, however it consistently decreased after 2000 time steps. After 10000 simulation time, inflammatory response vanished away. However, inflammatory response took different history for tissue at G6, which has strain lower than G4-1. As can be seen, the inflammatory response was lower compared to G4-1 (as expected). It decreased after 2000 simulation time, but increased afterward. This occurred several times as the whole system was trying to reach equilibrium. In the case of G4-1, the inflammatory response was consistently suppressed since high strain caused rapid reactions from immune cells in the model, hence the signaling cytokines (TNF and TGF) can be rapidly spread on the tissue space. This is not the case in G6 as the cytokines were not fully spread, and immune cells could not work as efficiently as the in G4-1.

Each of the developed models at organ, tissue and cellular levels has been qualitatively assessed from the results, and found to follow the correct trends and behavior. The organ model demonstrated pressure drops as expected in airway system [25]. The cellular model results of inflammation are comparable to typical inflammation course times [26]. It will be challenging to compare or validate quantitatively the integrated multi-scale model as the environment and developed models are very specific than those used in the literature for this study. Overall the results from the multi-scale models are representative and provide information that is observed in this specific application.

The model presented has certain limitations. These include the model only considers fluid mechanics until generation 7 of airways; the influence of lower airways was ignored. The tissue scale analysis was aimed to obtain strain value in each airway under consideration. The non-linear deformation of tissue was ignored in this study. In the cellular scale analysis, we simplified the inflammation process by considering a pro- and anti-inflammatory cytokines. There are more cytokines involved that may be influence the inflammation process. These needs be considered for accurate inflammation model. Table 5 summarized the model used in this study.

**TABLE 5:** Summary of the Multi-Scale Model

Level	Input	Output	Remarks
Organ (FSI)	Air flow time course during breathing	1. Pressure 2. velocity	Upper airway tissue experience elevated pressure.
Tissue (Structural Analysis)	Pressure	1. Stress 2. Strain	Strain of a single airway in lower generations are lower
Cellular (Agent-based model)	Strain	1. Inflammation score 2. cells spatial distribution	Higher strain leads to more severe injury, but also induce a better wound healing response

## IV. Conclusion

This study analyzed inflammation during mechanical ventilation by modeling it as a multi-scale system as inflammatory responses are complex processes that involve multiple scales ranging from cellular level to organ levels. The inflammatory responses from the cellular-level model, in turn, modulate changes in material properties at tissue and organ levels. Multiple models were developed at organ, tissue and cellular levels and integrated in a multiscale modeling framework. The CA model represented the cellular dynamics component while the plane-strain elasticity model was employed to account for tissue mechanics. The fluid-solid model effectively demonstrated the effect of inflammation from the lower scales to the pulmonary tree. A limitation of this study is that it used localized models for each component in modeling inflammation as a multiscale system. However, future research is planned to fully integrate the dynamics of each component in a multi-scale system.
